# Ethnic racial socialization within and across black immigrant and nonimmigrant families

**DOI:** 10.3389/fpubh.2026.1877193

**Published:** 2026-08-12

**Authors:** J’Mag Karbeah, Ifrah Abshir, Irma Hasham, Diana Augustin, Rainah Ross, Elizabeth Al, Charlize Ongeri, Ariam Aman

**Affiliations:** 1 Division of Health Policy and Management, University of Minnesota School of Public Health, Minneapolis, MN, United States; 2 Division of Epidemiology and Community Health, University of Minnesota School of Public Health, Minneapolis, MN, United States; 3 Department of Psychology, University of Minnesota College of Liberal Arts, Minneapolis, MN, United States; 4 Department of Social Work and Family Studies, St. Olaf University, Northfield, MN, United States

**Keywords:** black immigrants, ethnic-racial socialization, family socialization, parent child dyad, racial identity

## Abstract

**Introduction:**

Ethnic-racial socialization (ERS) is a protective mechanism used by Black parents to instill cultural pride and communicate information about race, ethnicity, and racism to their children. To date, few studies have examined the similarities and differences that exist in the messages that Black nonimmigrant and Black immigrant parents share. Additionally, little is known about whether discrepancies between parent and adolescent reports of ethnic racial socialization differ for Black immigrants and nonimmigrant families.

**Methods:**

The current study examines these potential differences through focus group interviews with 18 (7 Nonimmigrant, 11 immigrant) parents $\;\;({\bar{x}}_{\mathrm{age}}=41.1)$ and 16 (6 nonimmigrant and 10 immigrant) adolescent $\;\;({\bar{x}}_{\mathrm{age}}=15.0).$ Focus group sessions were conducted between June and November 2025, lasted 60–90 min, were audio-recorded, transcribed verbatim, and analyzed in NVivo using deductive and inductive codes from a collaboratively developed codebook. We leverage a concurrent integrative mixed-method design and present findings from a thematic analysis and content analysis of our focus groups.

**Results:**

The four deductive themes identified from our participants were: (1) ethnic-racial socialization messages; (2) coping socialization; (3) relational and contextual influences; and (4) outcomes and meaning making. We also present inductive themes that highlight socialization reporting discrepancies within and across immigrant and nonimmigrant dyads. ERS within immigrant and nonimmigrant Black families is dynamic, multifaceted, and contextually grounded.

**Discussion:**

These findings underscore the importance of recognizing variation in the ways Black families communicate and adolescent perceptions and responses to ERS messages. Interventions and supports aimed at improving adolescent mental health must account for these cultural nuances, building on family strengths to foster resilience and positive identity development among Black adolescents.

## 1 Introduction

For over four decades, multidisciplinary literature has examined the impact of ethnic-racial socialization, the behaviors and practices that educate adolescents about racial issues (1, 2). This process of socialization, especially the messages and tools that young people receive from their parents and caregivers, have been identified as protective against the psychological harm caused by racial discrimination (2, 3). Ethnic-racial socialization is a crucial aspect of Black child-rearing and the messages that Black children receive from their parents have been shown to have lasting impacts on intergroup interactions and psychosocial wellbeing (4). However, there is limited research examining ethnic variability within this process. Specifically, existing literature has explored socialization themes in samples of nonimmigrant Black American and Caribbean origin Black immigrant adolescents (5, 6), but few studies have examined this variability between nonimmigrant Black families and African origin Black immigrant families (7–10). The percentage of Black Americans with different ethnic and immigration histories complicates the ethnic-racial socialization processes that have been leveraged to make sense of and navigate the realities of Black identity within the United States (11). While extant research has found that both age and socializing agent impact the salience of socialization messaging, to our knowledge, few studies have looked at how adolescents from African immigrant families receive and appraise parent-driven socialization messages (12). Although the impacts of parental ethnic racial socialization have been documented for multiple decades, researchers often note that parent and adolescent reports of parenting practices may have points of convergence and divergence that may yield crucial insights regarding adjustment and family functioning (13). Researchers have explored these discrepancies within Black parent-adolescent dyads (14), and within groups of immigrant adolescents and young adults (7) but less is known about these discrepancies within African origin Black immigrant dyads.

### 1.1 Racial identity development among Black immigrants

Although the majority of Black Americans are multigenerational Black individuals whose ancestors came to the United States as enslaved people, recent studies suggest that the ethnic diversity of Black Americans has continued to increase significantly since the 1980s (15, 16). Approximately 20% of Black Americans have recent family ancestry outside of the United States and identify as either first-generation or second-generation Americans with at least one foreign-born parent (16, 17). Despite both being racialized as Black, this influx of Black immigrants into the US has resulted in tensions between nonimmigrant and immigrant Black communities (11, 18). Most immigrant Black individuals hail from either the West Indies or sub-Saharan Africa, locations with dramatically different sociopolitical histories and environments wherein race is not as salient as it is in the United States. In these postcolonial majority Black environments, race is considerably less salient than within the U. S. where a still contentious history of chattel slavery and Jim Crow shapes every aspect of life and economic mobility (18). Black immigrants as they attempt to adapt to the culture of their chosen destination often find the task of acculturating, the psychological process through which an individual changes as a result of encountering another cultural group (19, 20), to be especially complex. New arrivals often have to negotiate their position within an anti-Black racial hierarchy that was largely absent in their countries of origin. Acculturation occurs across multiple domains including but not limited to behavior and identity and is often marked by an immigrant’s psychological adjustment to their receiving nation (19, 21). Acculturation theoretical frameworks note that immigrants’ acculturation can often be assessed according to the degree of participation in their ethnic culture, the culture of the new country, and for Black immigrants, Black American culture (20). These three dimensions of acculturation capture the contextual realities of Black immigrants to the US. To navigate the complexities of being Black in America, Black immigrants have two options: to their turn to multigenerational Black Americans to navigate living in a anti-Black racial society, or to attempt to align themselves with Eurocentric dominant culture norms that often result in the embrace of anti-Black rhetoric (22–24). This tridimensional acculturation poses challenges for adolescents in Black immigrant families who navigate three cultures in ways that are unfamiliar to their parents and peers.

### 1.2 Ethnic-racial socialization (ERS) among youth from Black immigrant families

For immigrant adolescents and adolescents born into immigrant origin Black families, the process of racial identity formation, is marred by the complexities of race and immigration status within an anti-Black dominant racial hierarchy (11). Cultural stress theory asserts that the stress and strain among parents and their children caused by migration-related stressors, like acculturation, can lead to increased risk of adverse outcomes such as behavioral problems and increases in risk behaviors (25). As Black immigrant parents attempt to navigate new intercultural dynamics in their receiving country and as young people navigate three cultures, many tensions may arise between how parents hope their children will engage with Black American culture, dominant American culture, and their culture of origin. Black immigrant parents who may not feel connected or represented by Black American culture may encourage their adolescents to distance themselves from what they perceive to be a cultural out-group in attempts to stave off discrimination or stereotyping (7, 26, 27). This study and its focus on ethnic racial socialization within and across Black immigrant and nonimmigrant groups adds to the growing body of research in developmental psychology that has found ethnic-racial socialization to be protective for children living in contexts where experiences of discrimination due to race or ethnicity are common (3). This study expands upon this literature by acknowledging that the information that parents share with their children regarding racial and ethnic group membership may differ across immigrant and nonimmigrant families.

### 1.3 Parent-adolescent discrepancies in ethnic racial socialization

Existing literature on reports of parenting behavior notes that there are often notable points of convergence and divergence between adolescents and their parents (13). Adolescence is a developmentally complex period that is marked by expanding social relationships that have the potential to support positive or impede positive development. Developmentally, adolescents have advanced cognitive and critical thinking skills that when combined with their need for autonomy complicate how ethnic racial socialization messages are received and interpreted by young people. Cognitively, adolescents can critically assess socialization messages shared by their parents and may be more likely to endorse the socialization messages they receive based on their own experiences with peers. Existing studies examining discrepancies in parent and adolescent reports of socialization have found few discrepancies in reported levels of cultural socialization and mixed results regarding messages related to preparation for bias (14, 28). The growing body of literature focused on ERS messages among Black immigrants have looked at differences between socialization messages and how adolescents internalize these messages, however, this scant body of research has focused on ERS messages about nonimmigrant Black populations generally (7).

### 1.4 The current study

The purpose of the current study is to identify potential differences in ERS messages that Black immigrant parents share with their children and how these messages differ from the messages being communicated in Black nonimmigrant households. Noting the complex acculturative dynamics facing adolescents in Black immigrant families, we aim to highlight ERS a complex and dynamic process that takes on various forms within and across Black immigrant and nonimmigrant families while also highlighting points of concordance and divergence in socialization experiences as reported by parents and adolescents. The current study asked the following research questions:

1. Do the types of ERS messages Black parents share with adolescents differ across immigrant and nonimmigrant families?
2. What ERS messages do adolescents report receiving from their parents? Do these messages differ for immigrant and nonimmigrant Black families?

This study advances recent calls for scholarship on Black immigrant identity development to include qualitative data that expands upon a body of literature that to date has been largely quantitative (11). Additionally, our analysis of discrepant perceptions of ethnic racial socialization across parent-adolescent dyads provides valuable insights regarding how Black immigrant and nonimmigrant adolescents engage with ethnic racial socialization messages.

## 2 Methods

The current study uses an integrative mixed method (IMM) design to examine: (1) ethnic racial socialization practices within Black immigrant and nonimmigrant families; and (2) points of convergence and discrepancy within parental socialization that arise during interviews with families (29). The IMM approach is a concurrent mixed method design method that allows us to conduct both a thematic analysis and a quantitative content analysis of our focus group data with parent-adolescent dyads to understand the content and frequency of parent driven ethnic racial socialization in immigrant and nonimmigrant Black families. The research team conducted four in-person focus groups with Black immigrant and nonimmigrant parent-adolescent dyads between June 2025 to November 2025 in the Minneapolis- St. Paul metropolitan area. The University of Minnesota Institutional Review Board approved this work under STUDY00023907.

### 2.1 Participants

#### 2.1.1 Recruitment

The goal of recruitment was to maximize representation across of countries of origin and experiences within our Black immigrant and nonimmigrant participants. The study team distributed flyers broadly and engaged community partners directly to invite families into the study. However, to provide a sense of group cohesions, we conducted separate focus groups for our immigrant and nonimmigrant families. Additionally, to minimize cultural variation within each immigrant focus group, we explicitly recruited from a Somali focus group, a non-Somali East African focus group, and a West African focus group based on the size of populations present in the research area (30). Additional we hoped to create groups that had shared language or religious traditions to minimize variation within each focus group. Interested families were asked to email the study team email, at which point they were sent a brief screening form to assess eligibility (e.g., youth aged 13 to 18, both parent and child self-identified as Black or African American, English-speaking, could attend in person). Families who met all inclusion criteria were then asked to complete a short form to verify eligibility details and confirm their intent to participate. Once both forms were returned, eligible dyads were emailed and scheduled for the focus group with which they best aligned.

#### 2.1.2 Demographics

We ultimately conducted four focus groups: two nonimmigrant Black focus groups (7 parents and 6 adolescents across the two groups), one Somali focus group (8 parents and 8 adolescents), and one West African focus group (3 parents and 2 adolescents). Although recruitment aimed to enroll Black immigrant families from a range of countries of origin and experiences, we had difficulty recruiting a diverse immigrant sample. We had difficulty recruiting from non-Somali East African communities and West African communities despite considerable effort and community connections. Our sample of immigrant families includes only five non-Somali participants (3 parents and 2 adolescents), representing two different West African countries (i.e., Liberia and Gambia), which reflects the difficulty in recruitment we experienced. We were able to successfully recruit both male and female parents as well as male and female youth participants. Our parent and youth participants ranged in age and a more detailed breakdown of our population demographics can be found in Table 1. Because the goal of the current study is to identify differences between immigrant and nonimmigrant families, our Somali and West African participants were combined into a single Black immigrant group, contrasted with the nonimmigrant Black group.

**Table 1:** Demographic characteristics of sample.

Ethnicity	Parents	Youth	Total
Age ( ${\bar{X}}_{\mathrm{age}}$ )	41.1	15.0	28.8
Sex (%)			
Male	4 (22.2)	7 (43.8)	11 (32.4)
Female	14 (77.8)	9 (56.3)	23 (67.6)
Group			
Nonimmigrant	7 (38.9)	6 (37.5)	13 (38.2)
Immigrant	11 (52.4)	10 (47.6)	21 (61.8)

### 2.2 Data collection

We developed a semi-structured focus group guide to learn about: (1) ERS messages being shared by immigrant and nonimmigrant parents to their children; (2) the frequency and contexts in which socialization occurred; and (3) sources of convergence and discrepancy in parent and adolescent reports of ERS. In addition to the open-ended questions presented in our focus group guide, we also ended each focus group by asking both parent and adolescent participants whether they endorsed five ERS items. These five items are taken from the Hughes and Johnson (31) ethnic racial socialization scale to capture the concepts of cultural socialization, egalitarianism, promotion of mistrust, and preparation for bias. These items were: (1) *race does not matter;* (2) *with hard work you can achieve anything;* (3) *you should not trust white people;* (4) *you should be proud to be Black;* and (5) *you will experience discrimination because of your race/identity.* Respondents were asked to raise their hand if they agreed with the statement that had just been read out loud by the facilitator. Participants were given time after each statement to think before raising their hand in agreement. Within some, but not all groups, participants offered additional exposition regarding their choice to agree or disagree with each statement.

All four focus group sessions occurred in person. The first three sessions were conducted at an office building located on the campus of a large midwestern university that offered ample parking for study participants. Our final session was conducted at a location owned by a community partner which also included ample parking for participants. Each focus group lasted approximately 60–90 min, was conducted in English, and followed the semi structured interview guide. Parents and adolescents participated in separate groups that cooccurred. Sessions were facilitated by research assistants (IA, DA, RR, AA, CO) and were audio-recorded using zoom for transcription purposes. At the start of each session, facilitators reviewed study aims, procedures, and community ground rules. Participants selected pseudonyms which were stored in a separate location from identifying information. After participation, both parents and youth received a $100 gift card as a token of appreciation for their participation.

### 2.3 Positionality and reflexivity

Because discussions of ethnic-racial socialization are highly contextual and sensitive, we attended explicitly to our positionality as a research team. Our team includes members who share ethnic, racial, and immigrant-origin backgrounds with the communities studied, as well as members who do not. The PI is a Black woman who immigrated to the United States from West Africa during early childhood. The research team consisted of Black women, three of whom are African and Caribbean immigrants and one who identifies as a nonimmigrant Black person. In addition to this core research team, a final team member assisted with coding who identifies as a non-Black person of color. While the PI has also worked closely with organizations in the study area who work with Black adolescents from immigrant and nonimmigrant backgrounds but had fewer relationships with organizations that serve both adolescents and parents. This composition shaped our access to community partners, our rapport with participants, and our interpretation of the data. To surface and check our assumptions, team members kept reflexive notes during coding, discussed how personal experiences might shape interpretation, and used team adjudication led by the principal investigator to resolve divergent readings of the data.

### 2.4 Analysis

#### 2.4.1 Thematic analysis

This study leveraged deductive and inductive thematic analyses to understand the ethnic racial socialization messages that Black immigrant parents shared with youth and the messages that youth report receiving. We also identified strategies for discrimination shared by parents and strategies leveraged by our multiethnic youth sample. Lastly, we explored how socialization experiences influenced outcomes and meaning making within our sample. Our deductive analysis was informed by ethnic-racial socialization theory to code responses (32).

To ensure accurate coding, all members of the research team received training in qualitative research methodology from the University Survey and Assessment Services which covered both qualitative methodology and appropriate coding techniques. This training occurred over the course of two weeks. Members of the research team first read through the transcripts and made notes of responses related to socialization experiences and identified which aspect of the theory the response most aligned with. After an initial read through, team members (IA, DA, RR, CO) independently coded each transcripts. The team meet to discuss codes and create a preliminary codebook that included each form of socialization including cultural socialization, preparation for bias, and promotion of mistrust, and exposure to egalitarian messages (31). We also added an inductive acculturation code to capture how youth and families negotiate their culture of origin, Black American culture, and dominant U. S. culture (19, 20). Our complete coding scheme is outlined in the Appendix. In addition to socialization messages, the team also identified instances where respondents referred explicitly to coping strategies they had developed in response to discriminatory experiences and how they made meaning of these experiences. The research team created inductive codes to capture these coping experiences and themes related to meaning making. The preliminary codebook was used to code both parent and youth focus group transcripts. After preliminary coding, team members met to identify points of concordance and disagreement within their coding and identification of key themes. The principal investigator, JK, adjudicated disagreements in coding when necessary. A second round of coding was done by team member IH once adjudication occurred.

#### 2.4.2 Content analysis

To measure concordance and discrepancies in perceptions of ethnic racial socialization messages between parents and adolescents, we followed Neundorf’s guidelines for inductive approaches to content analyses. Coders used NVIVO software and the previously established code book used for thematic analysis to enumerate the frequency of each theme within parent and adolescent respondents. We report these descriptive frequencies across our nonimmigrant and immigrant groups in our analysis. Due to our sample size, we did not perform any statistical analyses to determine if there were statistically significant differences in the content of parent-adolescent or nonimmigrant-immigrant messages.

Consistent with the IMM methodology, the content analysis allows us to quantitatively assess dominant themes in the ethnic racial socialization experiences shared by our participants. These frequencies allow us to quantify whether the ERS messages that adolescents report receiving are aligned with the messages most frequently shared by parents.

## 3 Results

### 3.1 Descriptive statistics

The demographic characteristics of our sample can be seen in Table 1. Across all focus group we recruited 34 individuals, which included 18 parents and 16 adolescent. The average age of parents in our sample was 41.1 years, and 15.0 years for our adolescent participants. We had representation from both male and female parents and youth participants which added to the richness of our conversation. The majority, 67.6% of our sample was female. Our parent sample included three fathers (1 nonimmigrant). Within our immigrant participants, we had three parents and two youth who identified as West African and all other participants identified as Somali. In total the sample included 13 nonimmigrant Black participants and 21 immigrant-origin participants.

### 3.2 Thematic analysis

Our analysis identified and mapped to 4 overarching themes that captured the information shared by our participants: (1) Ethnic Racial Socialization Messages, (2) Coping Socialization, (3) Relational and Contextual Influences, and (4) Outcomes and Meaning-Making.

#### 3.2.1 Ethnic racial socialization messages

In this study we focus on four mechanisms of ERS, cultural socialization, egalitarianism, promotion of mistrust, and preparation for bias (31). This theme and associated subthemes were deductively derived and reflect themes present in existing literature on ERS. We introduce a fifth deductive theme, *acculturation,* which is a key feature of literature examining the experiences of adolescents in immigrant families (19, 20).

##### 3.2.1.1 Cultural socialization

Cultural socialization refers to how parents teach their children about racial and ethnic group membership and can include sharing historical narratives, speaking their native language at home, or engaging in cultural practices (33). Cultural socialization can shape interpersonal relationships within and across racial and ethnic backgrounds in ways that can have psychosocial benefit for children (34, 35). Parents and youth consistently described cultural socialization messages that instilled pride in heritage, language, and religion, alongside preparation for discrimination and bias. Across groups, these messages were routine parts of family life but also intensified around key moments (e.g., school transitions, travel, high-profile news). Mothers were often named as primary messengers of culture and pride, while fathers more frequently emphasized vigilance and risk. Somali participants tended to link racial/ethnic identity to religious practice (e.g., prayer, Qur’anic study, mosque/duxi attendance), whereas African American participants anchored pride in history, lineage, and representation. Cultural socialization functioned as a foundation for identity. Somali families emphasized religion, food, and language as everyday anchors of belonging (e.g., learning to pray alongside parents, Friday Mosque routines, community events that showcase Somali culture). Parents described intentionally “exposing” children early so that, by school age, they could clearly name who they are (Somali/Black), the language(s) they speak, and their faith. Nonimmigrant Black families emphasized intergenerational knowledge as teaching “the whole history,.” Including atrocities and triumphs to cultivate confidence and pride in Blackness, often situated in civil rights narratives and contemporary representation. These practices positioned pride as both protective and aspirational, reinforcing messages about capability and worth. One Somali parent summarized this emphasis on pride and self-definition: “So to me, what’s really important … is that my child should identify as being African or Black, and be proud of it.”

A non-Somali immigrant similarly stressed heritage and language as anchors of identity, reflecting, “Language, definitely. Because one thing I dislike is not growing up knowing my language, my whole dialect, like I only speak English.” Across groups, families showed concordance in treating cultural pride as a foundation of identity that they instilled early; the discordance lay in its anchor. Immigrant-origin families, and Somali families in particular, centered religion, language, and nation of origin, whereas nonimmigrant Black families centered lineage and civil rights history. This likely reflects the differing sociopolitical histories of postcolonial countries of origin versus a multigenerational U. S. context (18, 20).

##### 3.2.1.2 Preparation for bias

Consistent with findings from other studies, participants in our sample noted that being prepared for discriminatory encounters, or bias incidents was also seen as a crucial part of ERS. Within our non-immigrant parent focus groups, parents shared that they explicitly discussed their Black identity with their children to prepare them for bias experiences and increase their safety. One mother, Jessica noted, “… I would definitely say that I discuss being African American or Black in my household for safety reasons. I have four boys, so it’s certain way once you are outside of my household that you have to conduct yourself, because wither the police gonna kill you, or… you know,some messed up stuff, [and] you gonna end up in something [you] ain’t got no business.” This statement reflects the long history of racialized violence against Black Americans which is often at the forefront of parents’ minds as they try to teach their children about their Black identity.

Experiences of discrimination, while discussed in all focus groups, were especially nuanced in conversation with our Somali participants who often noted how the discrimination they experience was deeply influenced by their religious identity as well as their racial identity. Somali adolescents and parents recalled travel-related warnings and airport encounters that primed them for being misread or suspected (e.g., being stereotyped as “terrorists”).

##### 3.2.1.3 Promotion of mistrust

Also consistent with existing literature on ERS experiences of Black immigrant adolescents, mistrust sometimes extended toward nonimmigrant Black people. One immigrant adolescent, “I think my parents stereotype like Black Americans … my mama will be talking down about my Black friends, like if they are from the hood or have Black dads.” Across both of our immigrant adolescent groups, adolescents noted that they did not endorse the messages about nonimmigrant Black peers that their parents shared and were often cautious about internalizing or accepting absolutist messaging from their parents.

##### 3.2.1.4 Egalitarianism

Egalitarian messages that emphasize individual action and choices rather than systemic barriers that Black individuals may face, are often underreported by adolescents. These messages that emphasize the role of individual skills, have been documented across multiple ethnic groups and the developmental impact of this color-blind perspective has been mixed in empirical research (3). Within our sample, found that immigrant origin parents were most likely to emphasize colorblind rhetoric. Egalitarian messages from immigrant parents in our sample highlighted the shared humanity of all people and advised adolescents not to seek universal approval, while still acknowledging that U. S. racial realities may not reflect egalitarian ideals in practice. Parents described teaching children to hold both truths: affirm equality internally while remaining alert to racism externally. As one Somali parent reflected, “In the end everyone is equal. We are all humans”. This notion of shared humanity also extended to the ability to achieve success in the United States that was voiced by other immigrant participants. Notably, a West African parent voiced a similarly opportunity-focused, egalitarian outlook, “There are great things in America. That’s why you are here … So take the best part of America.” This statement appears to paint opportunities and access to resources as uniformly distributed. This presumed distribution ignores the documented racial inequalities in the United States that often limit educational achievement and economic success within minoritized communities. Egalitarian messages were less frequently identified by our nonimmigrant participants who may have more personal experiences with structural inequity than their immigrant counterparts.

Egalitarian messaging showed the clearest intergroup discordance: although voiced across groups, it was strongest among Black immigrant-origin participants, who also endorsed “race does not matter” far more often than nonimmigrant counterparts when asked if they endorsed specific ERS items. This is consistent with postcolonial, majority Black contexts of origin where race is less salient than in the United States (18, 19).

##### 3.2.1.5 Acculturation

When asked about how they balance their culture from their country of origin with dominant US culture and Black American culture, our immigrants had varying views regarding the difficulties associated with acculturation. A West African adolescent, who was born in Liberia, a country where English is the national language but where most people speak an English-based creole language, noted feel as though her identity was distinct from other Black peers and that she felt she could not act like her peers noting, “At the end of the day, I have to remember this is not my country… So it’s like, I have to think, man, you do not have the same lives [as nonimmigrant Black peers]. Things you would do I would not do it because I know where I’m from, you know, stuff like that, but yeah, keep it like that.” While this perceived separation among immigrant adolescents is not surprising, in our sample there was a notable lack of discussions around acculturation from our Somali adolescent participants. Somali parents noted the tensions between the values of their country of origin and American culture. A Somali mother noted that these tensions are particularly difficult for adolescents who have not had the chance to visit Somali and become entrenched in the culture saying, “…we are against certain things, and then here they push it. So if I do not go ahead of it and be like, hey, whether they understand or not, they just gotta know where I stand, then it’s easier for them to kind of at least understand, okay, this what my culture says, and this is what they are saying.” The Somali adolescents in this study did not raise these tensions but instead focused heavily on their cultural socialization and alignment with Somali cultural values.

#### 3.2.2 Coping socialization

In addition to familiarizing adolescents with their cultural identity and developing a sense of belonging, both parents and adolescent participants shared various instances wherein they had communicated appropriate responses to racially discriminatory experiences. Strategies ranged from active confrontation or engagement to withdrawal, reflecting both parental guidance and adolescent agency. These coping practices were often context dependent, shaped by the severity of the incident, who was present, and the perceived risks of response.

##### 3.2.2.1 Engagement coping

Several adolescents described moments of directly challenging racism and action-oriented coping and engagement experiences. Nonimmigrant Black participants in particular reported exposing discriminatory acts among peers or responding in ways that disrupted the status quo. Confrontation was described as one way to resist discrimination and reclaim dignity. The confrontations mentioned included speaking up, alerting teachers, or in some cases physical altercation. One adolescent recounted confronting a peer who used a racial slur and engaging in a physical altercation, later telling their father, “I did it for the culture.” Such accounts reflected the ongoing negotiation between risk and empowerment that steered adolescent responses to discriminatory experiences. This particular response highlights that through explicit and implicit cultural socialization and preparation for discrimination, some adolescents felt that it was imperative that they defend not only themselves, but their entire cultural group against discriminatory action.

##### 3.2.2.2 Disengagement coping

While some participants felt that their sense of cultural pride called upon them to directly confront discriminatory language and actions, others in our sample felt that engaging with nefarious actors was never appropriate. Some parents and adolescents felt that to stay safe, the best way to cope with bias incidents was to disengage or avoid any altercation. Somali adolescents noted compliance and withdrawal during profiling or surveillance encounters, with one sharing that they “just ignored it” because without evidence they would not be in trouble. This statement at its core reflects the internalization of egalitarian or colorblind rhetoric and the belief that the due process would be justly applied and prevent any additional undue harassment. Nonimmigrant Black adolescents in our sample described disengaging from hostile peers or online harassment, choosing to log off or walk away to prevent escalation. One Black nonimmigrant female adolescent participant Jakaya noted, “Oh yea, people are weird on the internet. Just ignore them. Smile and wave. Bye, bye.” These narratives highlighted that avoidance was not necessarily resignation, but often a calculated choice to preserve safety and wellbeing. Parents across groups coached composure, self-respect, and confidence. Nonimmigrant parents more often layered in vigilance and the anticipation of unfair treatment, an adaptive response to sustained structural discrimination than their immigrant counterparts when teaching their children how to cope with discrimination.

#### 3.2.3 Relational and contextual influences

The delivery and reception of ethnic racial socialization messages were shaped by the dynamics within families, the presence or absence of supportive community members, and the timing of significant events. These influences determined not only what was said, but also how and when conversations took place.

##### 3.2.3.1 Parent and child dynamics

When asked why they chose to discuss their racial or cultural identity with their children as frequently as they noted, parents emphasized that their own lived experiences influenced the ways they communicated with their children. Even within families, participants often noted that existing family dynamics often shaped how messages were shared and how often they were shared. For example, a nonimmigrant Black mother contrasted her approach with her husband’s, noting, “My husband has the conversation with the kids, but he has it in a totally different way, because his experience is definitely different from mine. He was incarcerated for about 15 years, so he is more direct. I am more softer”. Within immigrant families, parents described modeling behaviors, such as prayer or language use, as powerful forms of teaching that sometimes-surpassed verbal instruction. Adolescents reinforced these observations, describing mothers as more likely to teach pride and culture, while fathers often stressed caution or risk. These patterns illustrate how ethnic racial socialization messages are mediated by family roles, personal histories, and intergenerational differences. An immigrant adolescent participant drew a similar contrast between their parents saying,: “Every time I have an issue, I always call my mom because she will always bring herself down to me, like we are friends … stuff I would not tell my dad, I would tell my mom.” Both of these statements highlight that lived experiences and gender shape how parents engage in ethnic racial socialization and with whom adolescents attempt to make sense of racialized experiences.

##### 3.2.3.2 Community and peers

Community spaces were described as critical for reinforcing or complicating family socialization messages. Somali parents emphasized mosques and cultural gatherings as spaces where adolescents could see their identities affirmed and practiced collectively. One parent explained that attending Islamic studies (i.e., duqsi) and mosque events helped children meet peers who shared their culture and religion, which “made them proud of who they are.” Nonimmigrant adolescents, who in our sample did not note religion as a major source of socialization, often described non-familial locations and actors such as schools and sports teams as sites of exclusion or overt racism. Experiences in these spaces served as negative socialization experiences and adolescents pointed to what they perceived to be discriminatory coaching decisions or explicitly being targeted with slurs within school and athletic spaces. Peers were also seen as inconsistent sources of support, sometimes affirming cultural pride but at other times amplifying discriminatory attitudes. One immigrant origin adolescent participant specifically noted how school and community organizations served as sources of affirmation; explaining, “I was in [Muslim Student Association] MSA and then Student Union, and African and Black Student Union … they really helped us learn what Black is and how to treat racism.”

##### 3.2.3.3 Event-based timing

When asked specifically about when they engaged in conversations about racial or ethnic identity, across both immigrant and nonimmigrant groups, participants described ethnic racial socialization conversations as often tied to triggering events rather than daily occurrences. Somali adolescents noted that such talks became more frequent during travel or following encounters with profiling, with one describing how airport experiences prompted explicit lessons from parents. Nonimmigrant Black adolescents identified school-based incidents and high-profile racial events in the media as sparks for family discussions, such as peers using racial slurs or news coverage of police killings. These findings underscore that the timing of ethnic racial socialization is highly contextual, often reactive, and shaped by both personal and societal events. An nonimmigrant participant echoed this event-driven pattern, noting that talks tended to follow “something really heavy,” such as a big news story. Although parents may engage in these conversations more regularly, conversations following specific discriminatory events or high-profile events appear to be remembered most vividly by adolescents.

#### 3.2.4 Outcomes and meaning-making

A pivotal aspect of the experiences shared by both immigrant and nonimmigrant participants was how they processed and made meaning out of their socialization experiences. This final theme highlights the variety of ways that parental socialization efforts shaped how adolescents understood their identities and influenced their overall emotional wellbeing. These outcomes were clustered around two subthemes: identity development, and resilience and mental health.

##### 3.2.4.1 Identity development

For Somali adolescents, identity was described as inseparable from religious and cultural practices. Being Somali and Muslim were viewed as intertwined, with prayer, Qur’anic study, and community gatherings serving as visible markers of belonging. Parents emphasized that exposure to the culture from their country of origin at an early age ensured children had a strong sense of cultural pride. Nonimmigrant Black families focused on intergenerational history and the complexity of shifting racial classifications across time. One parent reflected, “My dad’s birth certificate read as negro. My mom’s colored, mine was Black, and my daughter was African American. We are all in the same family, but we have all these different classifications.” For nonimmigrant families, their identities were tied closely to the racial hierarchy and racial dynamics of the United States. For adolescents from immigrant families, acculturation and the associated tensions were a salient experience that was mentioned by many. One adolescent described moments of identity strain, such as being perceived as “too White” in language or mannerisms, revealing the tension of navigating authenticity in different contexts. The complex and dynamic process of identity development was also reflected in our focus group with one West African adolescent reflecting on how her views toward her immigrant identity had shifted over time saying, “Growing up, we really did not like being African … but now I’m so African,” underscoring how belonging evolved through community connection.

##### 3.2.4.2 Resilience and mental health

Messages also shaped how adolescents processed and withstood racism. Parents encouraged confidence and reframing, often positioning discrimination as external rather than internal. One African American parent explained, “I want her to know the whole history. I want her to know the atrocities, I want her to know the triumphs, because I need her to understand that when people are out here talking about we are not human, do not listen.” Adolescents similarly articulated reframing strategies, with some describing racism as a societal illness rather than a personal failing. At the same time, participants acknowledged the toll of constant exposure, describing feelings of exhaustion, anger, and longing to leave environments where they were consistently marginalized. Despite these strains, affirmations such as “at the end of the day you are going to be somebody” reflected a shared belief that pride and resilience were essential tools for navigating daily life. For some immigrant adolescents, resilience was entangled with immigration-related concerns; one explained, “At the end of the day, I have to remember this is not my country … if I do something I can get deported back to my country, which is very embarrassing.” This tension of perpetual otherness highlighted by immigrant origin adolescents calls to the forefront how although they may experience the same racialized experiences as nonimmigrant peers, their experiences are further complicated by the fact that they have roots in another country.

### 3.3 Content analysis

**Figure 1:**
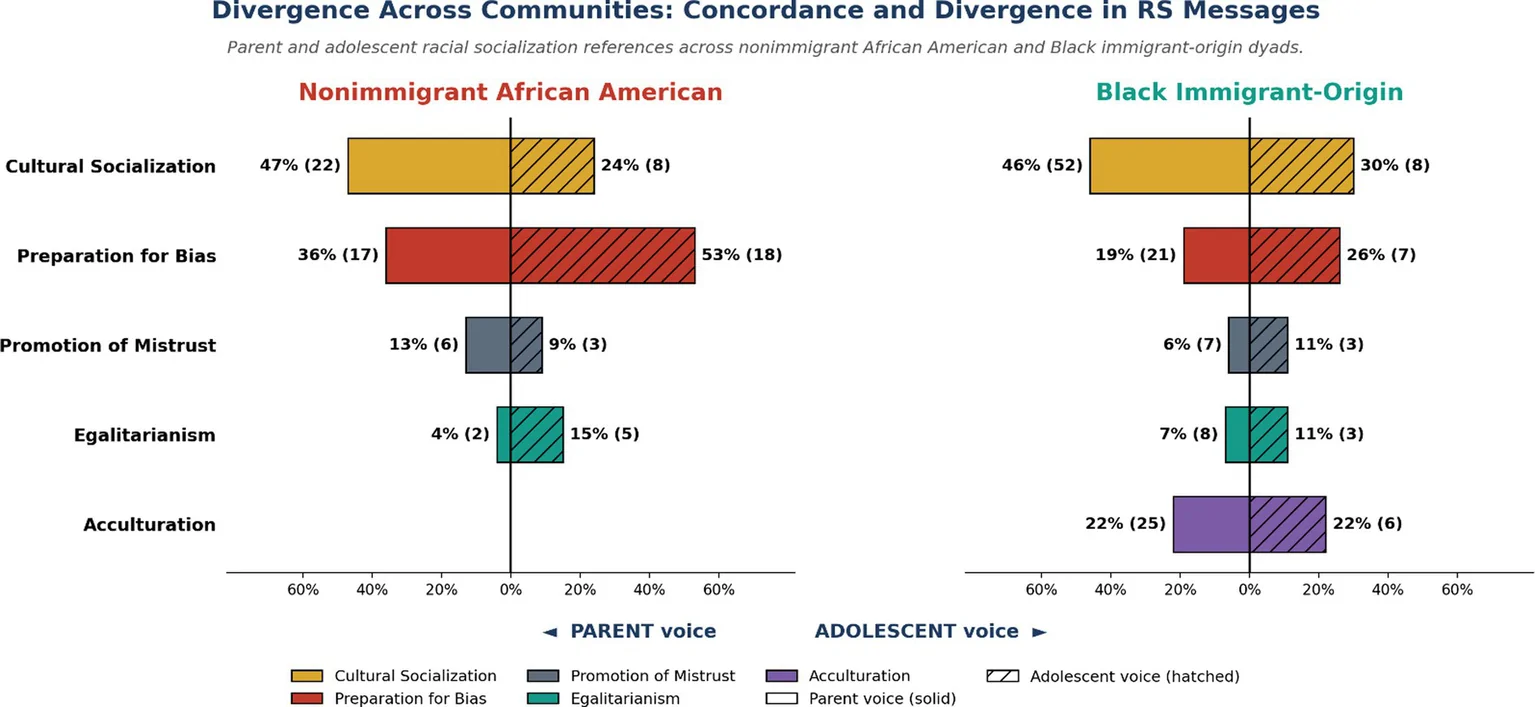
Frequency of ERS messages for parents and adolescents across nonimmigrant and immigrant participants.

The butterfly diagram in Figure 1 displays the findings of our content analysis and shows the frequency of each theme across parents and youth by nonimmigrant and immigrant group. Figure 1 focuses exclusively on reports of parent and youth reports of ERS.

Cultural socialization, and specifically cultural pride was the aspect of ERS most frequently noted by our immigrant respondents. Cultural socialization was the most frequently mentioned topic among nonimmigrant parents (47%) and immigrant parents (46%). These findings support the findings of our thematic analysis wherein participants focused on cultural pride as a central part of the conversations about race and ethnicity that happened in their households. Parents in both groups focused on positive cultural socialization and both noted that parents’ experiences and mothers often led conversations about cultural pride. An area of divergence among our groups that we can identify by revisiting our thematic analysis, are the mechanisms through which socialization occurs. Specifically, our immigrant parents noted that they used language as religion as ways to reinforce cultural identity and cultural pride compared to nonimmigrant parents who used historical examples to instill cultural pride.

Preparation for bias was the second most frequently communicated ERS theme among our nonimmigrant participants but not our immigrant respondents. In our sample, the salience of this preparation is a major source of divergence. Nonimmigrant parents shared messages promoting mistrust more often and often shared direct experiences of incarceration and profiling when talking to their children about bias and discrimination. Our immigrant parents were less likely to discuss preparing their children for bias. Only one in five statements from our immigrant parents focused on preparing adolescents for bias compared to nearly 40% of the comments shared by our nonimmigrant participants.

Promotion of mistrust and egalitarian messages were infrequently mentioned in both of our groups. Promotion of mistrust represented 13% of parent responses in our nonimmigrant group, and less than 10% of nonimmigrant adolescent responses. Similarly, only 6% of immigrant parent comments promoted mistrust and 11% of immigrant adolescent comments. Egalitarian messages constitute only 11% of immigrant adolescent responses and 15% of nonimmigrant adolescent response. Interestingly, youth were more likely to discuss egalitarian ERS messages than their parents in both groups.

## 4 Discussion

The diversity within the Black population in the United States allows for complex cultural in and out group dynamics wherein parents and youth across Black ethnic groups may share and receive ethnic racial socialization messages that reflect the unique social position of each group. In this study, we examined ethnic racial socialization messages across nonimmigrant and immigrant parents and adolescents. We explored how cultural identity shaped the frequency and content of messages shared with young people while also exploring the challenges that young people have when undergoing a tricultural acculturative process.

Across groups, we found strong alignment in the value families placed on cultural pride and socialization as well as the need to prepare adolescents for bias. In both groups cultural pride was and pride in Blackness was noted and affirmed. The salience of racial and cultural pride across groups indicates a shared unity among immigrant and nonimmigrant Black families that has not been identified in all previous work. Although our sample did have some instances wherein adolescents shared that their parents attempted to convince them that they were unlike their nonimmigrant peers, pride in being Black was shared by all participants which signals a level of unity within this diverse community. Nonimmigrant Black families grounded socialization in lineage, civil rights history, and structural preparation for bias, and more often promoted mistrust, consistent with a multigenerational experience of chattel slavery, Jim Crow, and continued institutional anti-Blackness (11). Black immigrant-origin families, and Somali families in particular, anchored pride in religion, language, and nation of origin and voiced a stronger egalitarian orientation, consistent with postcolonial, majority Black contexts where race is less salient than in the United States (18–20).

These qualitative patterns aligned with our quantitative content analysis. Immigrant-origin participants endorsed egalitarian statements far more often than African American participants, mirroring the qualitatively stronger egalitarian messaging in immigrant-origin families, while universal pride and near-universal preparation for bias appeared in both strands. At the same time, even nonimmigrant adolescents endorsed the mistrust statement at relatively low rates, echoing the qualitative finding that adolescents across groups pushed back against absolutist cautions. Our ability to quantitatively support the themes that emerged during our focus groups demonstrates the value of the mixed-method design for capturing how youth may be internalizing messages from their parents. Notably, youth mentioned experiences and examples of their parents preparing them for bias and discrimination after high-profile instances of racialized violence. This theme was mentioned much more frequently than parents who were more likely to discuss how they instilled cultural pride. Although being prepared for discriminatory encounters can be helpful for young people, research has shown that focusing exclusively on negative encounters may cause psychological distress for some adolescents (3). Although we did not ask participants in our sample how the messages they received impacted their mental health, it may be the case that youth associate their racial and ethnic identity with dangerous and potentially fatal outcomes because they remember more of these conversations than the affirming messages being shared by parents.

These findings carry practical implications. Mental health, school, and community programs serving Black youth should avoid treating Black families as a monolith and instead attend to ethnic, religious, and immigration-related variation in how families talk about race. Programs for immigrant-origin families might build on religious and cultural assets (e.g., partnering with mosques, duqsi, and cultural organizations) and prepare youth for religiously inflected bias such as Islamophobic profiling, while programs for African American families can validate vigilance and structural preparation. Because socialization conversations are largely event-triggered, schools and clinicians can anticipate that high-profile racial incidents will prompt family discussions and can offer timely, culturally responsive support.

This study has limitations. Our sample was small, drawn from a single midwestern metropolitan area, and unevenly distributed across countries of origin so within group nuances should be interpreted with caution. Sessions were conducted in English, which may have limited participation by less English-proficient family members. The quantitative strand was descriptive only and should be read as illustrative rather than confirmatory. Combining Somali and West African participants into a single immigrant-origin group aids comparison but obscures meaningful heterogeneity that future work should examine directly, ideally with larger, more balanced samples, multilingual data collection, and longitudinal designs that clarify how length of U. S. residence and generation shape these patterns.

Taken together, ethnic racial socialization within ethnically diverse Black families is dynamic, multifaceted, and contextually grounded. These findings underscore the importance of recognizing variation in the ways families communicate and youth internalize ERS messages, and they suggest that interventions and supports aimed at improving adolescent mental health must account for these cultural nuances, building on family strengths to foster resilience and positive identity development among Black youth.

## Data Availability

The raw data supporting the conclusions of this article will be made available by the authors, without undue reservation.

## References

[1] Hughes D Watford A Toro J. "A transactional/ecological perspective on ethnic–racial identity, socialization, and discrimination". In: Equity and Justice in Developmental Science: Implications for Young People, Families, and Communities [Internet] Eds. Horn, S., Ruck, M., and Liben, L. Cambridge, Massachusetts, USA: Elsevier Inc (2016). p. 41.

[2] Anderson RE Anyiwo N Gumudavelly D Reddy S Galán CA Nguyen AW . Racial socialization provision in African American and black Caribbean families. J Child Fam Stud. (2023) 32:1–16. doi: 10.1007/s10826-022-02415-w

[3] Neblett EW. Racial, ethnic, and cultural resilience factors in African American youth mental health. Annu Rev Clin Psychol. (2023) 19:361–79. doi: 10.1146/annurev-clinpsy-072720-015146 36854288

[4] Simon C. The role of race and ethnicity in parental ethnic-racial socialization: a scoping review of research. J Child Fam Stud. (2021) 30:182–95. doi: 10.1007/s10826-020-01854-7

[5] Lambert SF Rose T Saleem FT Caldwell CH. Ethnic-racial socialization, perceived neighborhood quality, and psychosocial adjustment among African American and Caribbean black adolescents. J Res Adolesc. (2021) 31:120–38. doi: 10.1111/jora.12586, 33070434

[6] Joseph N Hunter CD. Ethnic-racial socialization messages in the identity development of second-generation Haitians. J Adolesc Res. (2011) 26:344–80. doi: 10.1177/0743558410391258

[7] Thelamour B George Mwangi CA. “I disagreed with a lot of values”: exploring black immigrant agency in ethnic-racial socialization. Int J Intercult Relat. (2021) 85:26–36. doi: 10.1016/j.ijintrel.2021.09.002

[8] Guluma B. I’m not Habesha, I’m Oromo: immigration, ethnic identity, and the transnationality of blackness. Sociol Race Ethn. (2023) 9:486–501. doi: 10.1177/23326492231169250

[9] Ludwig B. It is tough to be a Liberian refugee in Staten Island, NY: the importance of context for second generation African immigrant youth. Afr Black Diaspora. (2019) 12:189–210. doi: 10.1080/17528631.2018.1559782

[10] Gibson BJ Exploring Generational Difference of Acculturation, Ethnic Identity and Racial Identity in Liberian Immigrants and their Children Living in the United States Western Michigan University (2021). Available online at: https://search.proquest.com/openview/d4410bebf4205891b33f754ce1006457/1?pq-origsite=gscholar&cbl=18750&diss=y&casa_token=yppChhWEbzUAAAAA:CNwg-7WXmRuMNJY2dmZImF4OrEtGvWvghjVkYYUEtXJUcrwaewtYAHKP0Xn-aaFhzTH9irpoig (Accessed June 11, 2025).

[11] Thelamour B Smith NA Tormala TT Hunter CD. Black immigrant racial identity scholarship in the United States: a conceptual examination. Identity. (2024) 25:486–500. doi: 10.1080/15283488.2024.2404504, 37339054

[12] Umaña-Taylor AJ Zeiders KH Updegraff KA. Family ethnic socialization and ethnic identity: a family-driven, youth-driven, or reciprocal process? J Fam Psychol. (2013) 27:137–46. doi: 10.1037/a0031105, 23421841 PMC3652612

[13] De Los Reyes A Ohannessian CM. Introduction to the special issue: discrepancies in adolescent–parent perceptions of the family and adolescent adjustment. J Youth Adolesc. (2016) 45:1957–72. doi: 10.1007/s10964-016-0533-z 27384957

[14] Christophe NK Jim S Rihal TK Gutierrez FJ Desmarais A Bañales J . Black parent-child ethnic-racial socialization reporting discrepancies and links with youth’s ethnic-racial identity. Child Dev. (2026) 97:532–45. doi: 10.1093/chidev/aacaf046, 41636556

[15] Piña G Cahn A Im C Key Findings about Black Immigrants in the US Pew Research Center (2026) Available online at: https://www.pewresearch.org/short-reads/2026/03/20/key-findings-about-black-immigrants-in-the-us/ (Accessed March 20, 2026).

[16] Tamir C Key Findings about Black Immigrants in the U.S Pew Research Center (2022) Available online at: https://www.pewresearch.org/short-reads/2022/01/27/key-findings-about-black-immigrants-in-the-u-s/

[17] Emeka A. “Just black” or not “just black?” ethnic attrition in the Nigerian-American second generation. Ethnic Racial Stud. (2019) 42:272–90. doi: 10.1080/01419870.2018.1427881

[18] Vickerman M. Black immigrants, perceptions of difference, and the abiding sting of blackness. J Am Ethnic Hist. (2016) 36:71–81. doi: 10.5406/jamerethnhist.36.1.0071

[19] Berry JW. Acculturation: living successfully in two cultures. Int J Intercult Relat. (2005) Special Issue: Conflict, negotiation, and mediation across cultures: highlights from the fourth biennial conference of the International Academy for Intercultural Research) 29:697–712. doi: 10.1016/j.ijintrel.2005.07.013

[20] Ferguson GM Iturbide MI Gordon BP. Tridimensional (3D) acculturation: ethnic identity and psychological functioning of tricultural Jamaican immigrants. Int Perspect Psychol. (2014) 3:238–51. doi: 10.1037/ipp0000019

[21] Clarke AV Poe CH Rose C Patel SG. "First- and second-generation black immigrant experiences with ethnic misidentification, acculturation, and psychological distress". In: Johnson DJ Chuang SS Glozman J, editors. Re/Formation and Identity: The Intersectionality of Development, Culture, and Immigration [Internet]. (2021) Cham: Springer International Publishing. p. 249–72.

[22] Hernandez KAC Murray-Johnson KK. Towards a different construction of blackness: black immigrant scholars on racial identity development in the United States. Int J Multicult Educ. (2015) 17:53–72. doi: 10.18251/ijme.v17i2.1050

[23] Agi AC Rivas-Drake D. “Other people’s battles”? Incorporating the experiences of immigrant-origin black American youth in ethnic/racial identity research. Identity. (2022) 22:101–15. doi: 10.1080/15283488.2021.1919518

[24] Alex-Assensoh Y. African Immigrants and African-Americans: An Analysis of Voluntary African Immigration and the Evolution of Black Ethnic Politics in America. Leiden, Netherlands: Brill. (2009)

[25] Salas-Wright CP Kagotho N Vaughn MG. Mood, anxiety, and personality disorders among first and second-generation immigrants to the United States. Psychiatry Res. (2014) 220:1028–36. doi: 10.1016/j.psychres.2014.08.045, 25223256 PMC4258138

[26] Coleman-King C. The (re-)Making of a Black American: Tracing the Racial and Ethnic Socialization of Caribbean American Youth. New York: Peter Lang (2014).

[27] Awokoya J. Identity constructions and negotiations among 1.5- and second-generation Nigerians: the impact of family, school, and peer contexts. Harv Educ Rev. (2012) 82:255–81. doi: 10.17763/haer.82.2.9v77p329367116vj

[28] Chen S Jelsma E Hou Y Benner A Kim SY. Antecedents and consequences of discrepant perceptions of racial socialization between parents and adolescents within Mexican-origin families. J Youth Adolescence. (2021) 50:2412–26. doi: 10.1007/s10964-021-01487-z, 34480295 PMC10319340

[29] Castro FG Kellison JG Boyd SJ Kopak A. A methodology for conducting integrative mixed methods research and data analyses. J Mix Methods Res. (2010) 4:342–60. doi: 10.1177/1558689810382916, 22167325 PMC3235529

[30] Tamir C Anderson M. Black Minnesotans | MN compass [internet]. (2026). Available online at: https://www.mncompass.org/chart/b14739-1/black-minnesotans (Accessed July 2, 2026).

[31] Hughes D Rodriguez J Smith EP Johnson DJ Stevenson HC Spicer P. Parents’ ethnic-racial socialization practices: a review of research and directions for future study. Dev Psychol. (2006) 42:747–70. doi: 10.1037/0012-1649.42.5.747, 16953684

[32] Braun V Clarke V. Using thematic analysis in psychology. Qual Res Psychol. (2006) 3:77–101. doi: 10.1191/1478088706qp063oa

[33] Wang Y Benner AD Kim SY. The cultural socialization scale: assessing family and peer socialization toward heritage and mainstream cultures. Psychol Assess. (2015) 27:1452–62. doi: 10.1037/pas0000136, 25961139 PMC4641843

[34] Tran AGTT Lee RM. Cultural socialization as a moderator of friendships and social competence. Cultur Divers Ethnic Minor Psychol. (2011) 17:456–61. doi: 10.1037/a0024728, 21767003

[35] Blackmon SM Coyle LD Davenport S Owens AC Sparrow C. Linking racial-ethnic socialization to culture and race-specific coping among African American college students. J Black Psychol. (2016) 42:549–76. doi: 10.1177/0095798415617865

